# Long-term results of a randomized phase III trial of TPF induction chemotherapy followed by surgery and radiation in locally advanced oral squamous cell carcinoma

**DOI:** 10.18632/oncotarget.4531

**Published:** 2015-06-19

**Authors:** Lai-ping Zhong, Chen-ping Zhang, Guo-xin Ren, Wei Guo, William N. William, Christopher S. Hong, Jian Sun, Han-guang Zhu, Wen-yong Tu, Jiang Li, Yi-li Cai, Qiu-ming Yin, Li-zhen Wang, Zhong-he Wang, Yong-jie Hu, Tong Ji, Wen-jun Yang, Wei-min Ye, Jun Li, Yue He, Yan-an Wang, Li-qun Xu, Zhengping Zhuang, J. Jack Lee, Jeffrey N. Myers, Zhi-yuan Zhang

**Affiliations:** ^1^ Department of Oral and Maxillofacial-Head and Neck Oncology, Ninth People's Hospital, College of Stomatology, Shanghai Jiao Tong University School of Medicine, China; ^2^ Department of Thoracic/Head and Neck Medical Oncology, University of Texas MD Anderson Cancer Center, Houston, TX, USA; ^3^ National Institute of Neurological Disorders and Stroke, National Institutes of Health, Bethesda, MD, USA; ^4^ Department of Oral Pathology, Ninth People's Hospital, College of Stomatology, Shanghai Jiao Tong University School of Medicine, China; ^5^ Department of Biostatistics, University of Texas MD Anderson Cancer Center, Houston, TX, USA; ^6^ Department of Head and Neck Surgery, University of Texas MD Anderson Cancer Center, Houston, TX, USA

**Keywords:** induction chemotherapy, oral squamous cell carcinoma, cisplatin, docetaxel, 5-fluorouracil

## Abstract

Previously, we conducted a randomized phase III trial of TPF (docetaxel, cisplatin, and 5-fluorouracil) induction chemotherapy in surgically managed locally advanced oral squamous cell carcinoma (OSCC) and found no improvement in overall survival. This study reports long-term follow-up results from our initial trial. All patients had clinical stage III or IVA locally advanced OSCC. In the experimental group, patients received two cycles of TPF induction chemotherapy (75mg/m^2^ docetaxel d1, 75mg/m^2^ cisplatin d1, and 750mg/m^2^/day 5-fluorouracil d1-5) followed by radical surgery and post-operative radiotherapy; in the control group, patients received upfront radical surgery and post-operative radiotherapy. The primary endpoint was overall survival. Among 256 enrolled patients with a median follow-up of 70 months, estimated 5-year overall survival, disease-free survival, locoregional recurrence-free survival, and distant metastasis-free survival rates were 61.1%, 52.7%, 55.2%, and 60.4%, respectively. There were no significant differences in survival rates between experimental and control groups. However, patients with favorable pathologic responses had improved outcomes compared to those with unfavorable pathologic responses and to those in the control group. Although TPF induction chemotherapy did not improve long-term survival compared to surgery upfront in patients with stage III and IVA OSCC, a favorable pathologic response after induction chemotherapy may be used as a major endpoint and prognosticator in future studies. Furthermore, the negative results observed in this trial may be represent type II error from an underpowered study. Future larger scale phase III trials are warranted to investigate whether a significant benefit exists for TPF induction chemotherapy in surgically managed OSCC.

## INTRODUCTION

The majority of patients with oral squamous cell carcinoma (OSCC) present with locally advanced disease [1, 2]. The routine recommendation for treatment of locally advanced OSCC is surgical management of the primary tumor and neck followed by post-operative radiotherapy or chemoradiotherapy depending on the presence of intermediate/high risk features [3, 4].

The use of induction chemotherapy prior to definitive treatment for management of locally advanced head and neck squamous cell carcinomas (HNSCC) is controversial [5-7]. In clinical trials TAX323 and TAX324, which investigated non-surgical management of HNSCCs, induction chemotherapy with docetaxel, cisplatin and 5-fluorouracil (TPF) was found to improve survival compared to induction cisplatin and 5-fluorouracil (PF) [8, 9]. Ghi et al. also recently reported an improvement in overall survival (OS) in patients treated with induction TPF followed by concurrent treatment (cetuximab/radiotherapy or PF/radiotherapy) compared to concurrent treatment upfront [10]. In contrast, the clinical trials DeCIDE and PARADIGM failed to demonstrate a benefit of TPF induction chemotherapy in HNSCC when given prior to concurrent chemoradiotherapy compared to chemoradiotherapy upfront [11, 12].

Our original randomized phase III trial of TPF induction chemotherapy in surgically managed locally advanced OSCC focused on OS as the primary endpoint. This study did not demonstrate an improvement in OS in patients that received induction TPF compared to surgery upfront [13]. In the current study, we report the long-term follow-up results of this trial.

## RESULTS

### Patients

**Figure 1:**
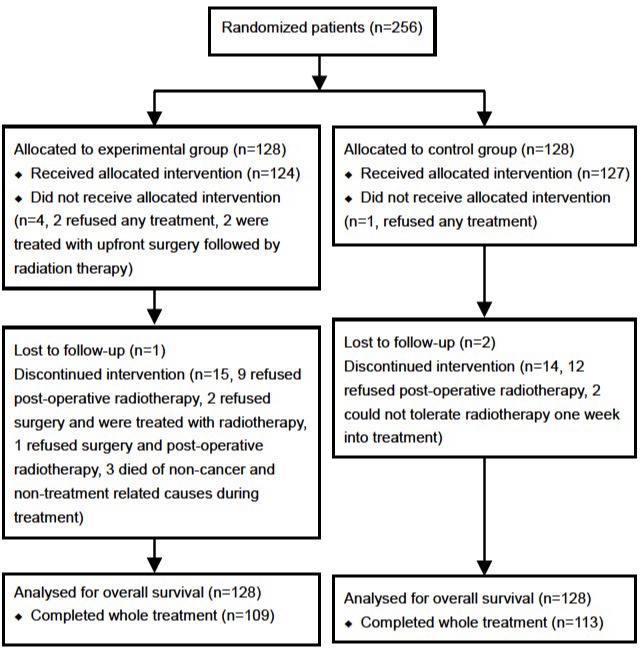
CONSORT diagram

As previously described, 256 patients were involved in this trial with 128 patients in each group. Five patients withdrew from this study, and 222 patients completed the full treatment protocol (113 patients in the experimental group and 109 in the control group) (Figure 1). Three patients were lost to follow-up. At the time of data cutoff in March 2015, the median follow-up time was 70 months.

### Survival analysis

**Figure 2:**
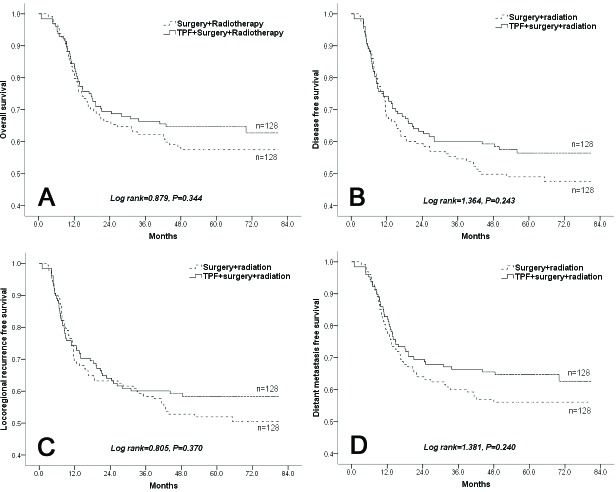
**Comparison of A.** overall survival, **B.** disease-free survival, **C.** locoregional recurrence-free survival, and **D.** distant metastasis-free survival in the experimental and control groups.

In total, the estimated 5-year OS rate was 61.1%, the estimated 5-year disease-free survival (DFS) rate was 52.7%, the estimated 5-year locoregional recurrence-free survival (LRFS) rate was 55.2%, and the estimated 5-year distant metastasis-free survival (DMFS) rate was 60.4%. There were no significant differences in OS, DFS, LRFS, or DMFS between the patients in the experimental group and control group (Figure 2).

### Pathologic response to induction chemotherapy

**Figure 3:**
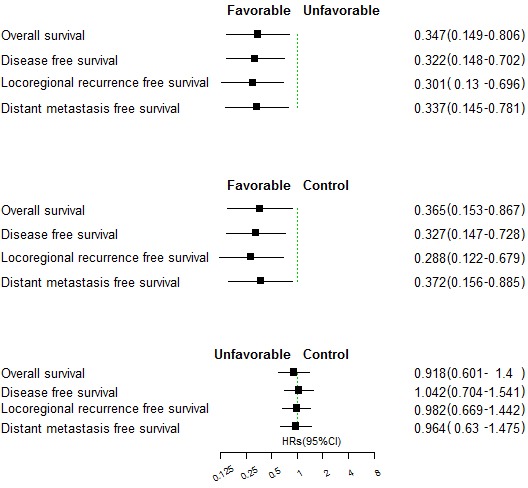
Comparison of overall survival, disease-free survival, locoregional recurrence-free survival and distant metastasis-free survival between the patients with favorable and unfavorable pathologic responses (upper panel), between favorable pathologic responses and the control group (middle panel), and between unfavorable pathologic responses and the control group (lower panel)

The favorable pathologic response rate to induction chemotherapy was 27.7%. Patients with favorable pathologic responses had a better OS, DFS, LRFS, and DMFS than those without favorable pathologic responses (HR = 0.365, 95%CI:0.153-0.867, *p* = 0.023 for OS; HR = 0.327, 95%CI:0.147-0.728, *p* = 0.006 for DFS; HR = 0.288, 95%CI:0.122-0.679, *p* = 0.004 for LRFS; HR = 0.372, 95%CI:0.156-0.885, *p* = 0.025 for DMFS). Furthermore, the patients with favorable pathologic responses also had a better OS, DFS, LRFS, and DMFS than those in the control group (HR = 0.347, 95%CI:0.149-0.806, *p* = 0.014 for OS; HR = 0.322, 95%CI:0.148-0.702, *p* = 0.004 for DFS; HR = 0.301, 95%CI:0.13-0.696, *p* = 0.005 for LRFS; HR = 0.337, 95%CI:0.145-0.781, *p* = 0.011 for DMFS). However, the differences in OS, DFS, LRFS and DMFS between the patients without favorable pathologic responses and the patients in the control group were not significant (Figure 3, Figure S1).

### Locoregional recurrence and distant metastasis

**Figure 4:**
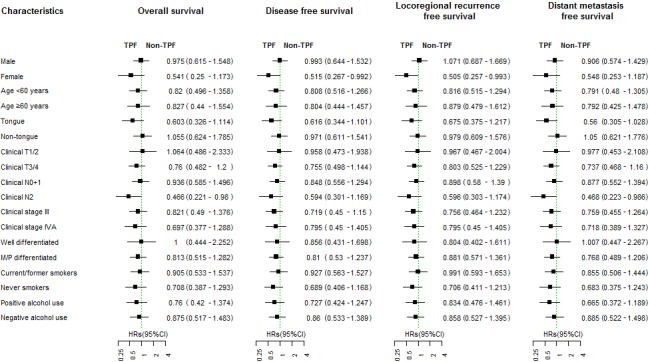
Subgroup analysis of overall survival, disease-free survival, locoregional recurrence-free survival and distant metastasis-free survival between the experimental and control groups

There was a trend towards a lower incidence of locoregional recurrence, distant metastasis, and secondary neoplasms for patients who received induction chemotherapy compared to upfront surgery; however, the difference was not significant (Table S1). The subgroup analysis showed no significant benefit from TPF induction chemotherapy in any of the subgroups, with the exception of cN2 patients, who seemed to have improved OS (HR = 0.466, 95%CI:0.221-0.98, *p* = 0.044) and DMFS (HR = 0.468, 95%CI:0.223-0.986, *p* = 0.046) as well as female patients, who had improved DFS (HR = 0.515,95%CI:0.267-0.992, *p* = 0.047) and LRFS (HR = 0.505,95%CI:0.257-0.993, *p* = 0.048) after TPF induction chemotherapy (Figure 4).

### Late adverse events (AEs)

No severe late AEs were found during the follow-up period of the patients.

### Surgical margins examined by immunohistochemistry

Among the 35 OSCC patients from 1985 to 1987, eight patients (22.9%) were found to have pan-cytokeratin positive surgical margins, which were previously examined and reported to be negative from tumor cells using hematoxylin and eosin staining. Among the 14 patients from 2000 to 2002 and 19 patients from 2008 to 2010, no patients were found to have pan-cytokeratin positive surgical margins.

## DISCUSSION

The long-term follow-up results of this trial demonstrated that TPF induction chemotherapy did not improve survival when compared to surgery upfront in OSCC. However, patients who received TPF induction and had a favorable pathologic response had a decreased risk for death and tumor recurrence compared to those who did not have a favorable pathologic response and those did not receive TPF induction chemotherapy.

This trial also underscores the importance of complete resection of the primary tumor including use of frozen sections during surgery to confirm adequate margins for R0 resection. Since our practice changed to include intra-operative margin evaluation in 1990s, there was a significant reduction in the incidence of possible residual tumor cells, using pan-cytokeratin-confirmed positive margins (22.9% in cases operated on from 1985 to 1987, versus 0 in patients operated on after 2000 and in this study). This could explain the increases in the 5-year OS rates in China from approximately 40% in the 1990s to 61% in the present study [14, 15]. R0 resection may play a key role in prevention of local tumor recurrence and thus contribute to improved OS. In regards to quality of life after R0 resection, immediate reconstructive surgery following tumor resection utilizing free flaps or pedicle flaps provides acceptable appearance and adequate oral function for OSCC patients at an otherwise late clinical stage.

Although OSCC patients treated with TPF induction chemotherapy did not have a significant increase in OS rates as a whole compared to those unexposed to induction chemotherapy, a trend towards improved survival was noted for those patients receiving induction chemotherapy. Previous meta-analyses have demonstrated that induction chemotherapy could reduce distant metastasis formation in patients with locally advanced HNSCC [5, 7]. In our study, patients receiving TPF induction chemotherapy had a 9%-higher 5-year DMFS than those not receiving TPF induction chemotherapy; this difference, however, was not statistically significant. Subgroup analysis demonstrated that cN2 patients receiving TPF induction chemotherapy had a better outcome than those not receiving TPF induction chemotherapy, especially in OS and DMFS. Therefore, induction chemotherapy could play a role in improving outcomes in patients at the highest risk of distant failure. As a result, a prospective trial of TPF induction chemotherapy in cN2 patients has been designed to confirm the subgroup analysis findings from this study (clinicaltrials.gov registered number of NCT02290145).

Patients with a favorable pathologic response to TPF induction chemotherapy had a more than 80% 5-year survival rate. In contrast, patients without a favorable pathologic response had similar outcomes compared to those patients who did not receive TPF induction chemotherapy. This may be very important in developing future personalized treatment strategies for OSCC. Along these lines, we have identified several proteins that could serve as predictive biomarkers in tissue specimens from patients who received TPF in this study, including Cyclin D1 and GDF15. cN2 patients with high Cyclin D1 expression exhibited significantly prolonged OS and DMFS after TPF induction chemotherapy, as did T3-4cN0 patients with high GDF15 expression [16, 17]. These two biomarkers have been selected as predictive biomarkers for response to TPF induction chemotherapy and are being prospectively evaluated in two ongoing induction chemotherapy clinical trials in OSCC (clinicaltrials.gov registered numbers of NCT02285543 and NCT02285530).

In conclusion, our long-term results do not support the use of TPF induction chemotherapy for all patients with locally advanced OSCC, receiving radical surgery followed by radiotherapy. However, specific patient populations defined by clinical and/or molecular criteria may benefit from induction TPF, and this selection strategy is currently being evaluated in prospective clinical trials with the ultimate goal of realizing personalized treatment protocols for this disease. In addition, the apparent lack of benefit for TPF induction chemotherapy, observed in this study may or may not be type II error secondary to an underpowered study. In the future, larger scale phase III trials are needed to determine whether there truly exists a role for TPF induction chemotherapy in the treatment of surgically managed OSCC.

## MATERIALS AND METHODS

### Patients and methods

The methodology of the original study has been previously published [13]. A redaction of the protocol is provided in the supplementary materials with a brief description provided here.

### Study design

This was a prospective, open label, randomized, phase III trial, which was approved by the institutional ethics committee at Ninth People's Hospital, Shanghai Jiao Tong University School of Medicine.

### Patients

Eligible patients included those 18-75 years of age with biopsy-confirmed, clinical stage III and IVA (T1-2N1-2M0 or T3-4N0-2M0, UICC 2002) disease and previously untreated OSCC. Patients were required to have resectable disease in the opinion of the treating surgeons. After eligibility was confirmed, patients were randomly allocated to the control group (surgery followed by post-operative radiotherapy) or experimental group (TPF induction chemotherapy followed by surgery and post-operative radiotherapy). Randomization without stratification factors was performed using sealed envelopes containing a computer-generated random number code.

### Treatment

Induction chemotherapy: in patients assigned to the experimental group, the palpable edges of the primary lesion (both the longest and shortest axes) were marked before induction chemotherapy by at least four points, which were 0.5cm away from the lesion. Chemotherapy consisted of docetaxel 75mg/m^2^, cisplatin 75mg/m^2^, and 5-fluorouracil 750mg/m^2^/day as a 120-hour infusion for 5 days. Induction chemotherapy was given every 3 weeks for 2 cycles. Surgery was performed at least 2 weeks after completion of induction chemotherapy.

Surgery: radical resection of the primary lesion and full neck dissection (functional or radical) with appropriate reconstruction (pedicle or free flap) was performed. The safety margins of the primary lesion were 1.5cm away from the palpable margins; for patients who received induction chemotherapy, the safety margins were 1.0cm away from the marks that were placed before induction chemotherapy to ensure the same extent of surgery in both groups. Frozen sections during surgery were performed to confirm adequate margins.

Post-operative radiotherapy: radiotherapy was initiated 4-6 weeks after surgery. Standard conformal or intensity-modulated radiotherapy was allowed at a dose of 1.8-2Gy/day, 5 days/week for 6 weeks, totaling 54-60Gy. In patients with high-risk features, a total radiation dose of 66Gy was recommended.

### Assessments

Clinical tumor responses were characterized according to the RECIST criteria version1.0 [18] at baseline and 2 weeks after cycle 2 of induction chemotherapy. Pathologic responses were assessed by examination of at least 20 slides of the resected specimen. A favorable response was defined as absence of any tumor cells or presence of scattered foci of a few tumor cells (minimal residual disease with < 10% viable tumor cells) [19]. Toxicities were assessed according to the CTCAE version3.0.

### Follow-up and outcomes

Patients were monitored every three months by physical examination in the first two years, every six months in the subsequent 3-5 years, and once a year thereafter until death or data censoring. Imaging was performed every six months. OS was calculated from the date of randomization to the date of death; DFS, LRFS, and DMFS were calculated from the date of randomization to the date of recurrence, locoregional recurrence, and distant metastasis or death from any cause, respectively.

### Detection of pan-cytokeratin expression in safety margins using immunohistochemistry

Serial sections from surgical safety margins were used to detect possible residual tumor cells in 68 OSCC patients (Table S2 and S3). 35 patients were randomly selected during 1985 to 1987, 14 patients were randomly selected during 2000 to 2002, and 19 patients were randomly selected from our trial during 2008 to 2010 (10 patients in the experimental group and 9 patients in the control group). Immunohistochemical staining was performed using well-established methods as previously described [16, 17] (details in the supplementary materials).

### Statistical considerations

For descriptive analysis, categorical data were expressed as number and percentage. The survival analysis was conducted using the Kaplan-Meier method and log-rank test. Hazard ratios (HR) were calculated using the Cox proportional hazards model. The intention-to-treat principle was applied for efficacy analysis. All hypothesis-generating tests were two-sided at a significance level of 0.05.

## SUPPLEMENTARY MATERIAL FIGURE AND TABLES


