# Supplementary material for: Long-term results of a randomized phase III trial of TPF induction chemotherapy followed by surgery and radiation in locally advanced oral squamous cell carcinoma
Source: Oncotarget. 2015 Jun 19;6(21):18707–14. doi: 10.18632/oncotarget.4531 (PMC4621922; doi:10.18632/oncotarget.4531)
Supplement: Supplementary file 1 [file oncotarget-06-18707-s001.pdf]

Long-term results of a randomized phase III trial of TPF induction chemotherapy followed by surgery and radiation in locally advanced oral squamous cell carcinoma

Supplementary Material

Pg. 2: Supplementary Materials and Methods

Pg. 3: Figure S1

Pg. 4: Table S1

Pg. 5: Table S2

Pgs. 6-8: Table S3

Pgs. 9-13: Study protocol redaction

SUPPLEMENTARY MATERIALS AND METHODS

Immunohistochemistry

Serial sections for each surgical margin, 4μm thick, were made using a sliding microtome and collected on silane-coated slides. Each margin was studied using hematoxylin and eosin (HE) and immunohistochemical staining for CKpan. HE sections were reviewed according to the WHO histological criteria [1]. The procedure of immunohistochemical staining was as follows: After deparaffinization with xylene, sections were transferred to water through ethanol. Before incubating in antibody solutions, sections were heated by water bath at 98°C with 0.01M citrate buffer solution (pH=6.0) for 20min to retrieve antigen, cooled at room temperature, and washed with phosphate buffer solution (PBS) 3 times for 5min each. Sections were then mixed with antibody solutions in following order: 3% hydrogen peroxide once for 5min, PBS 3 times for 2min each, mouse monoclonal antibody to pan-cytokeratin (dilution: 1/50) (Antibody Diagnostic Inc, USA) for 1h, PBS 3 times for 5min each, rabbit anti-mouse EnVision antibody (Dako Cytomation, Denmark) for 30min, and PBS 3 times for 5min each. The reactions were carried out in a moist box at room temperature. After incubation in the antibody solutions overnight at 4°C, reaction results were visualized by 3,3'-diaminobenzidine (DAB) detection kit (Dako Cytomation, Denmark) containing goat secondary antibody molecules against mouse immunoglobulin and DAB chromogen. Lastly, sections were washed with distilled water, counterstained by hematoxylin for 2min, washed with tap water and ethanol, and covered with coverslips. Negative controls were prepared by PBS instead of antibody.

Microscopic examination was performed by two pathologists and all samples were blinded. Both the sections with immunohistochemical staining and HE staining were examined to detect potential residual tumor cells in the margins. When positive staining against pan-cytokeratin was found in the submucosal layers, the relevant HE-stained sections were examined to confirm the positive margins.

Reference

1. Thompson L. World Health Organization classification of tumours: pathology and genetics of head and neck tumours. Ear, nose, & throat journal. 2006; 85(2):74.

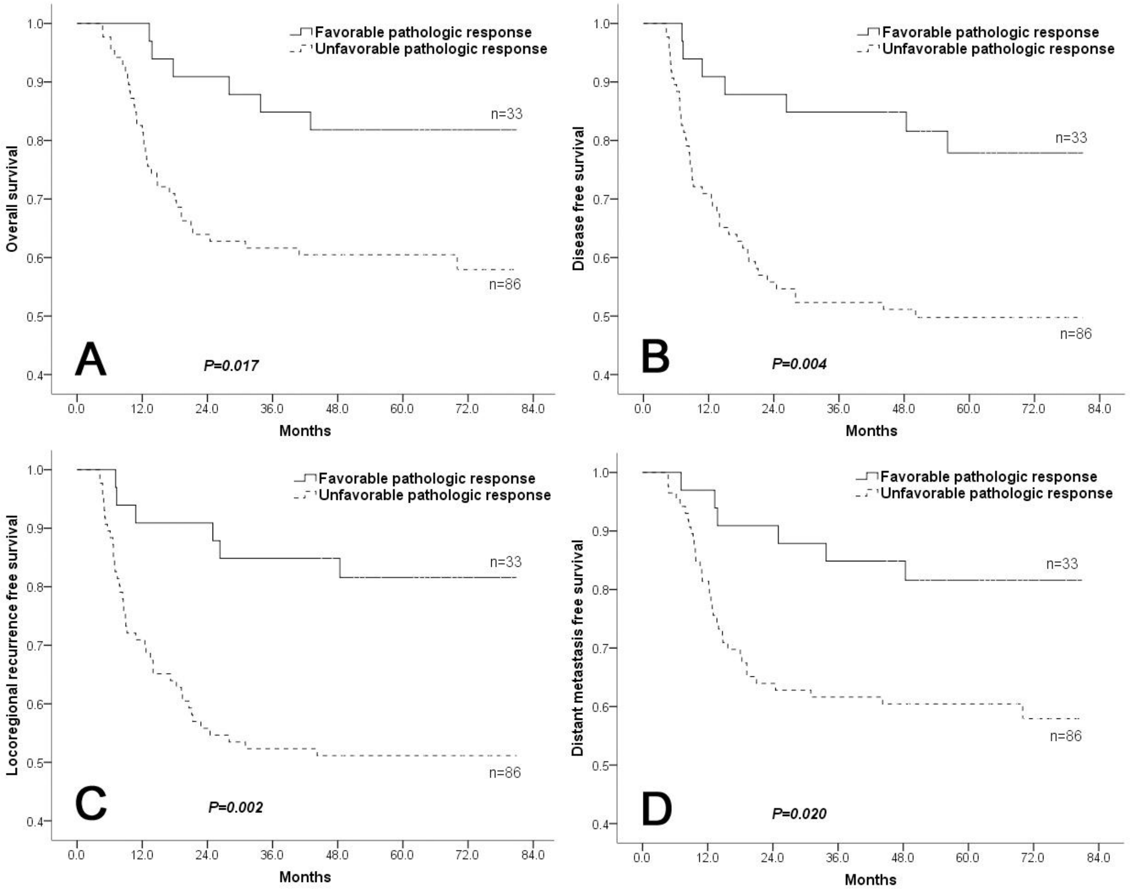

Figure S1. Comparison of (A) overall survival, (B) disease-free survival, (C) locoregional recurrence-free survival, and (D) distant metastasis free-survival between the patients with favorable and unfavorable pathologic responses. The landmark point was set at the time of response evaluation.

	Experimental group	Control group	Chi-square test value	P value
Locoregional recurrence	31.3% (40/128)	39.1% (50/128)	1.714	0.191
Distant metastasis	7.0% (9/128)	10.9% (14/128)	1.194	0.274
Secondary neoplasm	3.1% (4/128)	7.0% (9/128)	2.026	0.155

Table S1. Comparison of the incidences of locoregional recurrence, distant metastasis, and secondary neoplasm between experimental and control groups.

	Patients from 1985-1987	Patients from 2000-2002	Patients from 2008-2010
Gender			
Male	21	10	14
Female	14	4	5
Age (years)			
Range (median)	24-75 (55.2)	33-72 (55.9)	36-75 (56.1)
<60	20	9	13
≥60	15	5	6
Site			
Tongue	7	8	9
Buccal	8	1	2
Gingiva	14	2	3
Floor of mouth	3	1	2
Palate	3	1	2
Retromolar trigone	0	1	1

Table S2. Summary of 68 patients with oral squamous cell carcinoma for pan-cytokeratin expression detection in surgical margins.

Patient Number	Gender	Age (years old)	Site	Number of negative margins by hematoxylin and eosin staining	Number of positive margins by immunohistochemistry	Note
35 patients from 1985 to 1987						
1	Male	24	Tongue	4	0	
2	Female	27	Floor of mouth	2	0	
3	Male	34	Tongue	2	1	
4	Male	35	Tongue	5	0	
5	Female	42	Gingiva	5	0	
6	Male	42	Buccal mucosa	3	1	
7	Female	43	Buccal mucosa	3	0	
8	Female	44	Buccal mucosa	4	0	
9	Male	47	Palate	3	1	
10	Female	50	Gingiva	2	0	
11	Female	50	Tongue	3	0	
12	Female	52	Gingiva	3	0	
13	Male	52	Buccal mucosa	2	0	
14	Male	52	Floor of mouth	2	1	
15	Female	53	Gingiva	4	0	
16	Male	53	Gingiva	4	0	
17	Female	54	Gingiva	2	0	
18	Male	54	Buccal mucosa	2	1	
19	Female	58	Tongue	2	0	
20	Male	58	Gingiva	4	0	
21	Female	61	Palate	4	0	
22	Male	61	Gingiva	2	0	
23	Male	62	Gingiva	4	0	
24	Female	64	Gingiva	3	0	
25	Male	64	Floor of mouth	3	0	
26	Male	65	Buccal mucosa	3	0	
27	Male	65	Gingiva	5	0	

Table S3. Immunohistochemical results of pan-cytokeratin expression detection in the surgical margins from 68 patients with oral squamous cell carcinoma.

28	Male	66	Gingiva	3	0	
29	Male	67	Gingiva	4	1	
30	Female	69	Tongue	3	1	
31	Male	69	Buccal mucosa	4	0	
32	Male	72	Tongue	4	0	
33	Male	73	Palate	3	1	
34	Female	74	Gingiva	3	0	
35	Male	75	Buccal mucosa	6	0	
14 patients from 2000 to 2002						
1	Male	33	Retromolar trigone	4	0	
2	Male	45	Tongue	3	0	
3	Male	50	Tongue	5	0	
4	Male	53	Gingiva	4	0	
5	Female	54	Tongue	5	0	
6	Male	55	Floor of mouth	11	0	
7	Male	55	Tongue	3	0	
8	Female	58	Tongue	4	0	
9	Male	58	Gingiva	2	0	
10	Female	60	Tongue	3	0	
11	Male	60	Tongue	4	0	
12	Female	64	Tongue	5	0	
13	Male	66	Buccal mucosa	4	0	
14	Male	72	Palate	7	0	
19 patients from 2008 to 2010						
1	Male	36	Gingiva	4	0	
2	Female	45	Tongue	5	0	
3	Male	47	Tongue	9	0	
4	Male	48	Floor of mouth	4	0	TPF
5	Male	48	Tongue	3	0	TPF
6	Male	50	Tongue	11	0	TPF
7	Male	52	Tongue	5	0	
8	Male	53	Floor of mouth	5	0	TPF
9	Male	53	Tongue	5	0	
10	Female	54	Buccal mucosa	4	0	TPF
11	Male	57	Gingiva	5	0	TPF

Table S3 (continued).

12	Male	59	Palate	5	0	TPF
13	Male	59	Retromolar trigone	4	0	TPF
14	Female	61	Palate	5	0	TPF
15	Male	62	Tongue	4	0	
16	Male	67	Gingiva	4	0	
17	Female	69	Tongue	6	0	TPF
18	Male	70	Tongue	8	0	
19	Female	75	Buccal mucosa	5	0	TPF

Table S3 (continued).

STUDY PROTOCOL REDACTION

Introduction

Induction chemotherapy is regarded as an effective way to reduce or downgrade the locally advanced or aggressive cancers, and to improve the chance of eradication of the locoregional lesions by radical surgery and/or radiotherapy. However, there are still debates on the clinical value of induction chemotherapy for patients with advanced and resectable oral squamous cell carcinoma. Recently, an induction chemotherapy protocol of docetaxel, cisplatin and 5-fluorouracil (TPF) combination followed by chemoradiotherapy has been reported to be superior to cisplatin and 5-fluorouracil (PF) in two randomized phase III trials on the aspect of survival rate, and it is suggested as a better treatment strategy of induction chemotherapy for head and neck squamous cell carcinoma patients. However, there is still few direct evidence from large sample clinical trials on the survival rate, that confirming the benefit of TPF induction chemotherapy between the patients with and without TPF in oral squamous cell carcinoma (OSCC). The hypothesis of this study is that the induction chemotherapy of TPF protocol could benefit the patients with locally advanced OSCC.

Study design

This prospective, open label, parallel, interventional, randomized control trial was to evaluate the TPF induction chemotherapy in the patients with locally advanced and resectable OSCC. The patients would receive TPF induction chemotherapy followed by radical surgery and post-operative radiotherapy (the experimental group) or radical surgery and post-operative radiotherapy (the control group).

Primary endpoint: Survival rate.

Secondary endpoints: Local control and safety.

Sample size consideration

The study had a power of 83% on the basis of an assumed 5-year survival rate of 55% in the experiment group and 35% in the control group, with use of a two-sided log-rank test at a level of significance of 0.05. The recruitment period would be 3 years, and the follow-up period would be 3 years, and 15% of patients would drop out early or be lost to follow-up. A maximum of 128 patients per group were to be recruited.

Inclusion criteria

- Age: 18 to 75 years old.
- Sex: both males and females.
- Karnofsky performance status (KPS) >60.
- Histological biopsy confirming squamous cell carcinoma of the oral cavity (tongue, gingiva, buccal mucosa, floor of mouth, palate, and retromolar region).
- Clinical stage III/IVA (T1-2, N1-2, M0 or T3-4, N0-2, M0, UICC 2002) with resectable lesions.
- Adequate hematologic function: white blood cell >3,000/mm^3, hemoglobin>8g/L, platelet count>80,000/mm^3.
- Hepatic function: ALAT/ASAT <2.5 times the upper limit of normal (ULN), bilirubin <1.5 times ULN.
- Renal function: serum creatinine <1.5 times ULN.
- Written informed consent.

Exclusion criteria

- Evidence of distant metastatic disease and other cancers.
- Surgical procedure of the primary tumors or lymph nodes (except diagnostic biopsy).
- Previous radiotherapy or chemotherapy.
- Other previous malignancies within 5 years.
- Can not tolerate the treatment protocol with systematic diseases such as history of severe pulmonary or cardiac diseases.
- Legal incapacity or limited legal capacity.
- Creatinine clearance <30ml/min.

- Pregnancy (confirmed by serum or urine β-HCG) or lactation period.

Treatment procedures

The patients in the experimental group received the TPF induction chemotherapy for 2 cycles followed by radical surgery and post-operative radiotherapy. The palpable edges of the primary lesion (both the longest and shortest axis) were marked before induction chemotherapy by at least four points, which were 0.5cm away. The patients in the control group received the radical surgery and post-operative radiotherapy.

Induction chemotherapy: For the patients who were randomly assigned to receive TPF induction chemotherapy, peripherally inserted central catheter was firstly inserted before intravenous infusion, docetaxel (at a dose of 75mg/m^2 of body surface area) was administered as a 2-hour intravenous infusion, followed by intravenous cisplatin (75 mg/m^2), administered during a period of 2 to 3 hours. Then, 5-Fu (750 mg/m^2/day) was administered as a 120-hour continuous intravenous infusion for 5 days. Induction chemotherapy was given every 3 weeks for 2 cycles, unless there was disease progression, unacceptable toxic effects, or withdrawal of consent by the patients. Dexamethasone was given before docetaxel infusion to prevent docetaxel-related hypersensitivity reactions, skin toxic effects, and fluid retention; prophylactic antibiotics were also given starting on day 5 of each cycle for 3 days. Hydration with diuretic and antiemetic treatment was also performed. Primary prophylaxis with recombinant granulocyte colony-stimulating factor was not suggested. Chemotherapy dose reductions were allowed for grade 3/4 toxicities occurring after cycle 1: 25% and 50% dose reductions of the three chemotherapy agents were suggested for grade 3 and grade 4 hematologic toxicities or gastrointestinal toxicities, respectively; 25% and 50% cisplatin dose reductions were suggested for grade 3 and grade 4 renal toxicities, respectively. Surgery was performed at least 2 weeks after completion of induction chemotherapy.

Surgery: Radical resection of the primary lesion and full neck dissection (functional or radical) with proper reconstruction (pedicle or free flap) were performed. The safety margins of the primary lesion were 1.5cm far away from the palpable margins of the lesion; for patients who received induction chemotherapy, the safety margins were 1.0cm away from the marks that were

placed before induction chemotherapy, to ensure the same extent surgery in both arms. Frozen sections during surgery were performed to confirm adequate margins.

Post-operative radiotherapy: Radiotherapy was arranged 4 to 6 weeks after surgery. Routine external beam radiotherapy, such as conformal or intensity modulated radiotherapy was performed, and the dose was 1.8-2 Gy/day, 5 days/week for 6 weeks, and totally 54-60 Gy, in the patient with high risk features, such as positive surgical margin, extra capsular nodal spread, vascular embolism, total dose of 66 Gy was suggested.

Assessment

A complete medical history was obtained and tumor assessment was performed at baseline. Clinical tumor response was assessed by clinical evaluation and imaging study and was characterized according to the criteria of response evaluation criteria in solid rumors (version 1.0) before surgery. Post-operative pathologic response was assessed by post-operative pathologic examination as good and bad response. A good response was defined as absence of any tumor cells (pathologic complete response) or presence of scattered foci of a few tumor cells (minimal residual disease with <10% viable tumor cells); otherwise, a bad pathologic response was defined. Toxic effects were assessed weekly during and after completion of induction chemotherapy and radiotherapy according to the common terminology criteria for adverse events (version 3.0).

Outcome measures

Overall survival was calculated from the date of randomization to the date of death; disease free survival was calculated from the date of randomization to tumor recurrence or distant metastasis or death from any cause; locoregional recurrence/distant metastasis free survival was calculated from the date of randomization to locoregional recurrence/distant metastasis of tumor or death from any cause. Time to locoregional recurrence/distant metastasis was calculated from the date of finishing treatment to tumor locoregional recurrence/distant metastasis. Patients were

monitored by every three months in the first two years, every six months in the next 3 to 5 years, and once a year thereafter until death or data censoring.

Study chair: Zhi-yuan Zhang, Department of Oral & Maxillofacial-Head & Neck Oncology, Ninth People's Hospital, School of Medicine, Shanghai Jiao Tong University, China. Telephone: +86-21-23271699-5385. Email: zhang.z.y@hotmail.com.

Contact: Lai-ping Zhong, Department of Oral & Maxillofacial-Head & Neck Oncology, Ninth People's Hospital, School of Medicine, Shanghai Jiao Tong University, China. Telephone: +86-21-23271699-5160. Email: zhonglp@hotmail.com.

Study center: Ninth People's Hospital, Shanghai Jiao Tong University School of Medicine.

Study initiation: Jan. 1st, 2008.

Study completion (expected): Dec. 31th, 2015.

Study reviewed and approved by ethics Committee, Ninth People's Hospital, Shanghai Jiao Tong University School of Medicine, with the approval number of 2008[12].
